# Comparison of very high-power short-duration and ablation index guided catheter ablation for cavotricuspid isthmus block

**DOI:** 10.1007/s10840-026-02330-w

**Published:** 2026-04-18

**Authors:** Bettina Kirstein, Martin Benedikt, Charlotte Eitel, Jan-Per Wenzel, Sascha Hatahet, Tugba Aktemur Özalp, Christian-Hendrik Heeger, Roland Richard Tilz

**Affiliations:** 1 Department of Rhythmology, University Heart Center Lübeck, University Hospital Schleswig-Holstein, Lübeck, Germany; 2 Division of Cardiology, University Heart Center Graz, Medical University of Graz, Graz, Austria; 3 Department of Rhythmology, Cardiology and Internal Medicine, Asklepios Klinik Hamburg Altona, Hamburg, Germany; 4 German Center for Cardiovascular Research (DZHK), Partner Site Lübeck, Lübeck, Germany

**Keywords:** Typical atrial flutter, Cavotricuspid isthmus ablation, Radiofrequency, Very high-power short-duration, efficacy, safety, rhythm outcome

## Abstract

**Background:**

Catheter ablation for the treatment of typical atrial flutter (AFL) provides effective and durable cavotricuspid isthmus (CTI) block associated with encouraging clinical outcome. Safety and efficacy of very high-power short-duration (vHPSD) temperature-controlled radiofrequency (RF) CTI ablation with 90 watts over 4 s (90 W/4 s) for typical AFL in comparison to conventional contact-force (CF) ablation-index (AI) guided ablation remains insufficiently investigated.

**Methods:**

This single-center observational cohort study based on prospectively collected registry data included 100 consecutive patients undergoing CTI ablation for symptomatic typical AFL, with 50 patients treated using a vHPSD strategy (vHPSD-group) and 50 patients using an AI-guided ablation approach (AI-group).

**Results:**

All CTIs were successfully blocked using either method. Median RF applications and RF times in the vHPSD-group and AI-group were 36 [IQR 26;47] versus 18 [IQR 12;26], *p* < 0.001 and 142 s [IQR 105;189 s] versus 574 s [IQR 401;983], *p* < 0.001, respectively. No differences in periprocedural complications were observed. Recurrence of AFL after 12 months was low with no significant differences between groups (2% vs. 2%, *p* = 1). In patients with re-ablation there was no difference in CTI block durability between both groups (75% vHPSD-group versus 67% AI-group, *p* = 0.809).

**Conclusions:**

Very HPSD ablation (90 W/4 s) is a safe and effective strategy for CTI ablation, offering the advantage of markedly shorter CTI RF application times compared with AI-guided ablation. Rates of periprocedural complications, acute and durable CTI block, and long-term arrhythmia recurrence are similar between the two approaches.

**Condensed Abstract:**

We compared very high-power short-duration (vHPSD, 90 W/4 s) versus ablation-index-guided cavotricuspid isthmus (CTI) ablation in typical atrial flutter. Acute bidirectional CTI block was achieved in all patients. vHPSD significantly reduced CTI radiofrequency time without increased complications and showed similarly low recurrence and CTI block durability at 12 months.

**Graphical Abstract:**

**Figure:**
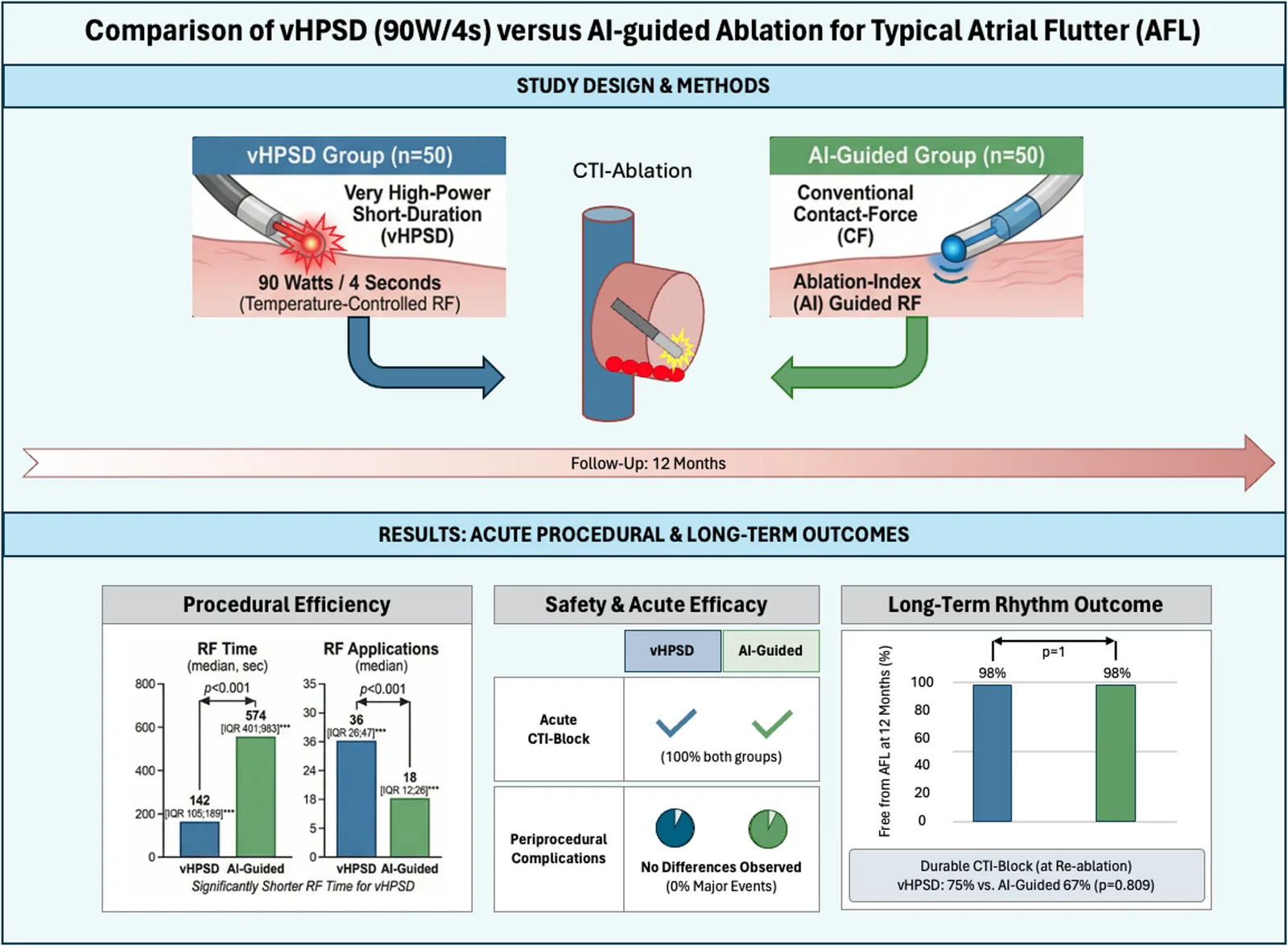

## Introduction

Typical atrial flutter (AFL) is the most common macro-reentrant atrial tachycardia, characterized by a reentrant electrical circuit that involves the cavotricuspid isthmus (CTI) [1, 2]. Catheter ablation (CA) of the CTI has become the gold standard of care for the treatment of symptomatic typical AFL, with high acute success rates and favorable long-term outcomes [3, 4].

Conventional CTI ablation typically uses moderate-power (30–50 watts), longer duration (20–40 s per lesion) radiofrequency (RF) energy applications, guided by parameters such as contact force (CF) and Ablation Index (AI). However, this approach can be time-consuming, may increase procedural burden and steam pop formation [5, 6].

Very high-power short-duration (vHPSD) temperature-controlled ablation, using 90 watts over 4 s per lesion (90 w/4 s), has recently emerged as a promising strategy in pulmonary vein isolation (PVI) for the treatment of atrial fibrillation (AF) [7–11]. Its use in PVI ablation has been explored with the rationale of creating effective, shallow, and wide lesions while minimizing collateral tissue injury and significantly reducing RF application time [12–14].

Currently, limited clinical data from small cohorts and short- to mid-term follow up suggest that vHPSD ablation may achieve comparable acute success and short-term rhythm outcomes in CTI ablation, while offering advantages in procedural efficiency [15]. In addition, evidence regarding the performance of this strategy in real-world cohorts including patients undergoing concomitant atrial fibrillation ablation remains scarce.

The present study therefore aimed to compare vHPSD CTI ablation with a conventional AI-guided approach in a contemporary clinical cohort and to evaluate procedural characteristics, safety, and rhythm outcomes with extended follow-up up to 12 months.

## Methods

### Study design and population

This study represents a single-center prospective, non-randomized, observational cohort study including consecutive patients undergoing CTI ablation for typical AFL at our institution during the study period. Clinical, procedural, and follow-up data were prospectively collected in an institutional ablation registry to evaluate efficacy, safety, and long-term rhythm outcome of vHPSD-CTI ablation for typical AFL in comparison to a conventional power-controlled CTI ablation protocol guided by the AI. Patients were treated using either ablation strategy according to operator preference and catheter availability in routine clinical practice. In all cases, CTI ablation was performed due to documented symptomatic AFL. Additional PVI for the treatment of concomitant documented symptomatic atrial fibrillation was performed if present and indicated.

Between September 2020 to December 2023 patients aged ≥ 18 years with documented symptomatic typical AFL scheduled for CTI ablation were included. Exclusion criteria were prior CTI or right atrial ablation, prior PVI or left atrial ablation, congenital heart disease, predominant atypical flutter, severe valvular heart disease, contraindication to post-interventional oral anticoagulation, switch to conventional mode in vHPSD CTI-ablation procedures, pregnancy, or inability to provide informed consent.

All patients provided written informed consent and all patient information was anonymized. The study was approved by the local ethics board (Lübeck ablation registry ethical review board number: WF-028/15) and performed in accordance with the ethical standards laid down in the 1964 Declaration of Helsinki and its later amendments.

### Peri-procedural management & ablation procedure

All patients underwent pre-ablation investigation as of the center’s standard of care. In brief, intracardiac thrombi were ruled out using transesophageal echocardiography. In patients on vitamin K antagonists, the procedure was performed under therapeutic INR values of 2–3. In patients on direct oral anticoagulants (DOAC) the morning dose was omitted on the procedure day. All ablations were performed under deep sedation using propofol, midazolam and fentanyl following the recommendations of a position paper by the German Cardiac Society [16]. Ultrasound-guided right femoral vein punctures (3 × 8Fr short sheaths) were performed and continuously flushed with heparinized saline (10 ml/h). One decapolar diagnostic catheter was introduced and positioned in the coronary sinus for reference and atrial activation mapping. In patients with AF undergoing PVI, an SL1 sheath was available for transseptal access, but its use during CTI ablation was at the operator’s discretion, as the procedure is confined to the right atrium.

### vHPSD ablation group

Ablation was performed using an open-irrigated tip catheter (QDOT MICRO™, Biosense Webster) in vHPSD mode (QMODE+), delivering 90 watts for 4 s per lesion with a minimum contact force of 15–20 g. Interlesion distance was defined at 3–4 mm with overlapping and closely timed (< 10 s) adjacent applications. The technique of short interlesion distance has been described for PVI in more detail before [10].

### AI-guided ablation group

Ablation was performed using an open-irrigated tip catheter (Thermocool Smart-touch SF, Biosense Webster) with AI guidance, targeting AI values of 450–500 along the CTI using 30–40 W of RF energy with a minimum contact force of 5 g. Maximum interlesion distance was 6 mm.

In both groups, three-dimensional electroanatomical mapping was performed using the Carto^®^ 3 system (Biosense Webster, Inc.). Lesions were delivered point-by-point guided by catheter stability and force criteria (VISITAG™ Module). CTI ablation was performed immediately after completion of PVI. The detailed intraprocedural management for 3D mapping, vHPSD and AI-guided PVI has been described previously [8]. The procedural endpoint was the achievement of complete bidirectional CTI block, confirmed by differential pacing maneuvers and electrogram analysis. Additional ablation lesions were applied as needed. Switch to the conventional mode in the vHPSD-group led to exclusion. All procedures in both groups have been performed by highly experienced operators only.

### Postprocedural care

A figure-of-eight suture, and a pressure bandage were used to prevent femoral bleeding. The pressure bandage was removed after four hours, and the figure-of-eight suture on the next day. Following ablation, all patients underwent transthoracic echocardiography immediately post procedure, after two hours and at post-ablation day one to rule out a pericardial effusion. Oral anticoagulants were re-initiated six hours post ablation. Anticoagulation was continued for at least three months and continued thereafter based on the individual CHA_2_DS_2_-VASc score. In patients with PVI, symptoms related to ETI were assessed post ablation and daily until discharge. Proton-pump inhibitors were orally administered once daily for 6 weeks post ablation post-PVI as part of the center´s standard of care. Post-procedural antiarrhythmic drug (AAD) therapy was limited to AF patients undergoing additional PVI and discontinued after the 3-month blanking period. Patients with sole AFL ablation did not receive routine AAD therapy. Re-initiation of AAD therapy and/or redo ablation was guided by patient symptoms and preference. In cases of AFL recurrence, redo CTI ablation was performed or considered the preferred approach.

### Endpoints and follow-up

The primary endpoint was acute procedural success, defined as achievement of bidirectional cavotricuspid isthmus (CTI) block.

Secondary endpoints were total CTI-RF time and freedom from typical atrial flutter (AFL) recurrence at 12 months, assessed by clinical follow-up, 12-lead ECG, and Holter monitoring at 3, 6, and 12 months. Recurrence was defined as any documented AFL episode. For AT/AF, recurrence was defined as any episode occurring beyond the 3-month blanking period. Periprocedural complications were documented during hospitalization and at each follow-up visit. CTI-specific metrics, including RF delivery time and number of RF applications, are reported separately. Total procedural time and total dose-area product reflect the entire procedure, including concomitant PVI when performed.

### Statistical analysis

Continuous variables were tested for normal distribution using the Shapiro-Wilk test. In case of normal distribution, data are presented as mean ± standard deviation (SD), otherwise as median and interquartile ranges (IQR): first quartile [Q1] and third quartile [Q3]. Comparison of continuous data was performed using the student’s t-test if normally distributed, otherwise with the Wilcoxon signed-rank test.

Categorical data are reported as absolute (n) and relative frequencies (%) and were compared using the chi-square test or Fisher’s exact test (small, expected frequencies). Statistical significance of the observed difference was expressed in terms of p-values. Two-sided p-values < 0.05 were considered statistically significant. All calculations were performed with the statistical analysis software DATAtab e.U. Graz, Austria.

## Results

### Patient characteristics

A total of 100 patients with symptomatic documented typical AFL underwent CTI ablation with either vHPSD 90 W/4 s (*n* = 50) or conventional CF-sensing AI guided ablation (*n* = 50). Patients were predominantly men (71%) at a mean age of 70 years with a median CHA_2_DS_2_Vasc Score 3 and preserved left ventricular ejection fraction. Concomitant symptomatic AF requiring PVI was frequent in both groups but significantly higher in the vHPSD-group which accounted for a significant higher AAD usage in this group at baseline (42% vs. 22%, *p* = 0.032). Co-morbidities were equally distributed, however type 2 diabetes mellitus was statistically more common in the conventional AI-group. Prior betablocker medication was frequently used in both groups. (76% each). Oral anticoagulation was established in all patients. Detailed patient baseline characteristics are shown in Table 1.

**Table 1:** Baseline demographic data

Parameter	vHPSD	AI-guided	*p*-value
Number of patients (n)	50	50	
Age (years), mean ± SD	72 [62; 76]	72 [65; 78]	0.441
Female gender, n (%)	18 (36)	11 (22)	0.123
Hypertension, n (%)	28 (56)	32 (64)	0.414
Diabetes mellitus, n (%)	4 (8)	12 (24)	0.029
Coronary artery disease, n (%)	18 (36)	15 (30)	0.603
Heart failure (any), n (%)	10 (20)	8 (16)	0.603
Left ventricular ejection fraction (%)	55 [52; 60]	58 [55; 60]	0.245
CHA₂DS₂-VASc score, median [IQR]	3 [1;4]	3 [2;4]	0.635
Concomitant atrial fibrillation, n (%)	46 (92)	23 (46)	< 0.001
History of stroke/TIA, n (%)	6 (12)	6 (12)	1
Anticoagulation use, n (%)	50 (100)	50 (100)	1
Prior betablocker use, n (%)	38 (76)	38 (76)	1
Prior antiarrhythmic drug use, n (%)	21 (42)	11 (22)	0.032

Continuous data are given in median and interquartile range (IQR) [Q1; Q3] or mean ± standard deviation (SD). Categorial data are given as percentages of n. AI: ablation index, vHPSD: very high-power short-duration, TIA: transitory ischemic attack

### Periprocedural characteristics

Procedural data are summarized in Table 2. Although predefined, no conversions from vHPSD to conventional ablation occurred, and all procedures were completed with the initially selected strategy. All CTIs were successfully blocked in either group. In the vHPSD-group, twice as much RF applications than in the conventional AI-guided group were needed for complete CTI ablation (36 [IQR 26;47] vs. 18 [IQR 12;26], *p* < 0.001). However, the median RF time for CTI ablation was four times shorter in the vHPSD-group (142 [IQR 105;189] vs. 574 [IQR 401;983] sec, *p* < 0.001). In the vHPSD-group, total procedure time including PVI when performed, was shorter and dose area product was lower, but without statistical significance.

**Table 2:** Procedural characteristics, periprocedural complications and rhythm outcome

Parameter	vHPSD	AI-guided	*p*-value
CTI-RF time (seconds)	142 [105; 189]	574 [401; 983]	< 0.001
CTI-RF applications (n)	36 [26; 47]	18 [12; 26]	< 0.001
Acute blocked CTI, n (%)	50 (100)	50 (100)	1
Total procedure time (minutes)*	70 [60; 96]	76 [45; 110]	0.767
Total procedure dose area product (cGy cm^2^)*	298 [210; 422]	334 [174; 568]	0.549
Periprocedural complications, n (%)	2 (4)	2 (4)	1
Any arrhythmia recurrence at 12 months, n (%)	11 (22)	5 (10)	0.171
AFL recurrence at 12 months, n (%)	1 (2)	1 (2)	1
AT/AF recurrence at 12 months, n (%)	10 (20)	5 (10)	0.262
Re-ablation, n (%)	4 (8)	3 (6)	0.695
Blocked-CTI at redo, n (%)	3 (75)	2 (67)	0.809

Continuous data are given in median and interquartile range [Q1; Q3] or mean ± standard deviation. Categorial data are given as percentages of n. CTI: cavotricuspid isthmus; RF: radiofrequency; AFL: atrial flutter; AF: atrial fibrillation. * Data reflect the entire procedure, including concomitant PVI when performed

### Safety

There was no difference in terms of major periprocedural complications (Table 2). In each group one patient experienced a transient sinus arrest. In the vHPSD-group, one patient developed hemodynamically non-relevant pericardial effusion which was managed conservatively. No pericardial aspiration or pericardial drainage occurred. One patient in the AI group experienced suspected gastric aspiration during procedural sedation and received prophylactic postprocedural antibiotic therapy. The patient remained clinically stable without oxygenation impairment, pneumonia, or septic complications. Continuous intraprocedural ECG monitoring was performed in all cases, and no significant ST-segment alterations or ischemic changes were observed. There were no further major complications such as major bleeding, major groin complications, cardiac tamponade, or stroke in either group. There were no documented steam pops. Clinically, no patient reported other postprocedural complications during the follow-up.

### Rhythm outcome

After 12 months, recurrence due to any atrial arrhythmia was numerically higher in the vHPSD group than in the AI group (22% vs. 10%, *p* = 0.171) but did not reach statistical significance. This was mainly driven by cases of AT/AF recurrences (20% vs. 10%, *p* = 0.262). Recurrence of AFL was low and did not differ between both groups (2% vs. 2%, *p* = 1).

All patients with AFL recurrence underwent redo ablation. In the AI group, one patient with combined AFL and AF/AT recurrence on Holter-ECG was found to have a non-durable CTI block and underwent successful redo ablation. Two patients with prior sole AFL and newly diagnosed persistent AF underwent re-ablation, both demonstrating durable CTI block. In the vHPSD group, one patient with isolated AFL recurrence was found to have a non-blocked CTI at redo ablation. Three patients with AFL and/or AT/AF recurrence, all with prior persistent AF, also underwent redo procedures, with CTI block confirmed in all cases.

Two patients in the AI group and seven patients in the vHPSD group with predominantly former persistent AF and paroxysmal AF/AT recurrence did not undergo redo ablation or restart AAD therapy because of low oligosymptomatic arrhythmia burden. In these patients, CTI block durability was not reassessed, and no remapping data were available.

## Discussion

### Main findings

We evaluated efficacy, safety, and rhythm outcomes in symptomatic patients with typical AFL undergoing CTI catheter ablation with vHPSD (90 W/4 s) compared with standard CF-sensing AI-guided ablation. The major findings are (1) Acute CTI block was achieved with both ablation strategies; (2) vHPSD (90 W/4 s) required two times more CTI-RF applications but markedly reduced CTI-RF time; (3) no major differences were observed regarding periprocedural complications or CTI block durability in the limited subset of patients undergoing redo procedures; and (4) AFL rhythm outcome at 12 months were similar between groups, though differences may be influenced by concomitant PVI.

### Current evidence of vHPSD-CTI ablation in typical AFL

Recent studies have evaluated the use of vHPSD ablation (90 W/4 s) for CTI ablation in typical AFL. Our findings are consistent with previously published results by Schillaci et al., confirming significant reduction in total RF time (Schillaci: 88 ± 40 s vs. 492 ± 269 s; *p* < 0.001; present cohort: 158 ± 80 s vs. 767 ± 509 s; *p* < 0.001) without evidence of compromised procedural safety or acute lesion efficacy [8]. Short- to mid-term follow-up in that study (≤ 8 months) demonstrated high acute success rates and low recurrence (vHPSD 4% vs. conventional 3%) [8].

In our cohort, extended follow-up to 12 months showed AFL recurrence rates of 2% in both groups (*p* = 1), and acute bidirectional CTI block was consistently achieved. Although only a small subset of patients underwent redo procedures, CTI conduction block durability appeared preserved irrespective of energy delivery strategy, though these numbers preclude firm conclusions regarding lesion durability.

Beyond confirming these long-term outcomes, our study provides incremental data in relation to prior work (e.g., Schillaci et al.). Importantly, a similar catheter platform (QDOT Micro) and comparator (Thermocool SmartTouch SF with AI guidance) have already been evaluated, and key procedural metrics such as RF delivery time have previously been reported.

Instead, the main contributions of the present analysis are: (1) a longer follow-up duration (12 months), allowing a more robust assessment of lesion durability; and (2) the inclusion of additional CTI-specific procedural details, particularly the number of RF applications. In addition, our cohort reflects a real-world population with a substantial proportion of patients undergoing concomitant AF ablation.

Taken together, these findings complement existing evidence by extending follow-up and refining procedural characterization, while supporting the overall safety and efficiency profile of vHPSD CTI ablation. These results should be interpreted as confirmatory and additive rather than indicative of procedural advance.

### Anatomical and lesion geometry considerations

The CTI is a highly variable substrate for ablation, with wall thickness ranging from < 1 mm to > 7 mm and frequent anatomic challenges such as sub-Eustachian recesses and pectinate muscle bundles [17, 18]. These features contribute to variable lesion formation and may necessitate multiple applications or alternative energy strategies.

Very HPSD creates wide, shallow lesions compared with conventional moderate-power longer-duration applications [12, 14]. Resistive heating dominates over conductive spread, which may limit lesion depth in thicker CTI sectors. Preclinical and clinical work suggests that lesion contiguity rather than single-spot depth becomes the key determinant of durable conduction block with vHPSD [19–21]. Shortening the inter-lesion distance (ILD) to approximately 3–4 mm markedly improves line continuity and reduces reconnection risk, since overlapping lesions achieve additive thermal effects and greater effective depth [21]. Closely timed adjacent or double RF applications (inter-lesion timing < 10 s) can further enhance lesion depth via thermal summation [19, 20]. Wider spaced lesions (> 5–6 mm) increases the likelihood of residual gaps, particularly in anterior or thickened regions.

In practice, CTI ablation with 90 W/4 s is feasible and efficient, although additional applications may be required to achieve complete line formation. Optimal technique includes tailoring inter-lesion distance to local CTI morphology, ensuring stable contact force, and consider overlapping or repeat applications with shorter inter-lesion time < 10 s in difficult regions, while monitoring for steam-pop risk. Thorough verification of bidirectional block remains essential, as anatomic heterogeneity means some areas may still require conventional longer-duration lesions and conversion to the conventional ablation mode, which is still possible with current catheter designs. Whether these considerations translate into long-term lesion durability is uncertain, as only a limited number of patients in both cohorts underwent redo ablation procedures. Observed CTI block rates in these cases were high for both strategies, but numbers are insufficient to draw definitive conclusions. Consequent re-mapping studies would be needed to assess lesion durability over time.

### Efficacy

The short per-lesion application time (4 s) with vHPSD enables rapid lesion creation, markedly reducing RF delivery time for CTI ablation in comparison to AI-guided ablation. Although more RF applications for CTI ablation were required, effective bidirectional CTI block was consistently achieved. Overall procedure times tended to be shorter with vHPSD, although differences did not reach statistical significance. This efficiency may facilitate workflow in electrophysiology laboratories, particularly when CTI ablation is performed in conjunction with other ablation procedures.

A recent study using a different catheter platform and HPSD protocol (TactiFlex, 50 W/12s) demonstrated higher first-pass bidirectional isthmus block rates and shorter procedure times compared with moderate-power ablation [22]. While that study primarily highlighted the acute procedural advantages of HPSD ablation, the present investigation evaluates a different catheter platform and a vHPSD protocol with markedly shorter application duration. Importantly, our study also provides long-term rhythm follow-up data and includes a real-world cohort, with a substantial proportion of patients undergoing concomitant AF ablation, thereby extending the available evidence by demonstrating the durability and practical applicability of this strategy in routine clinical practice.

### Safety

**Fig. 1:**
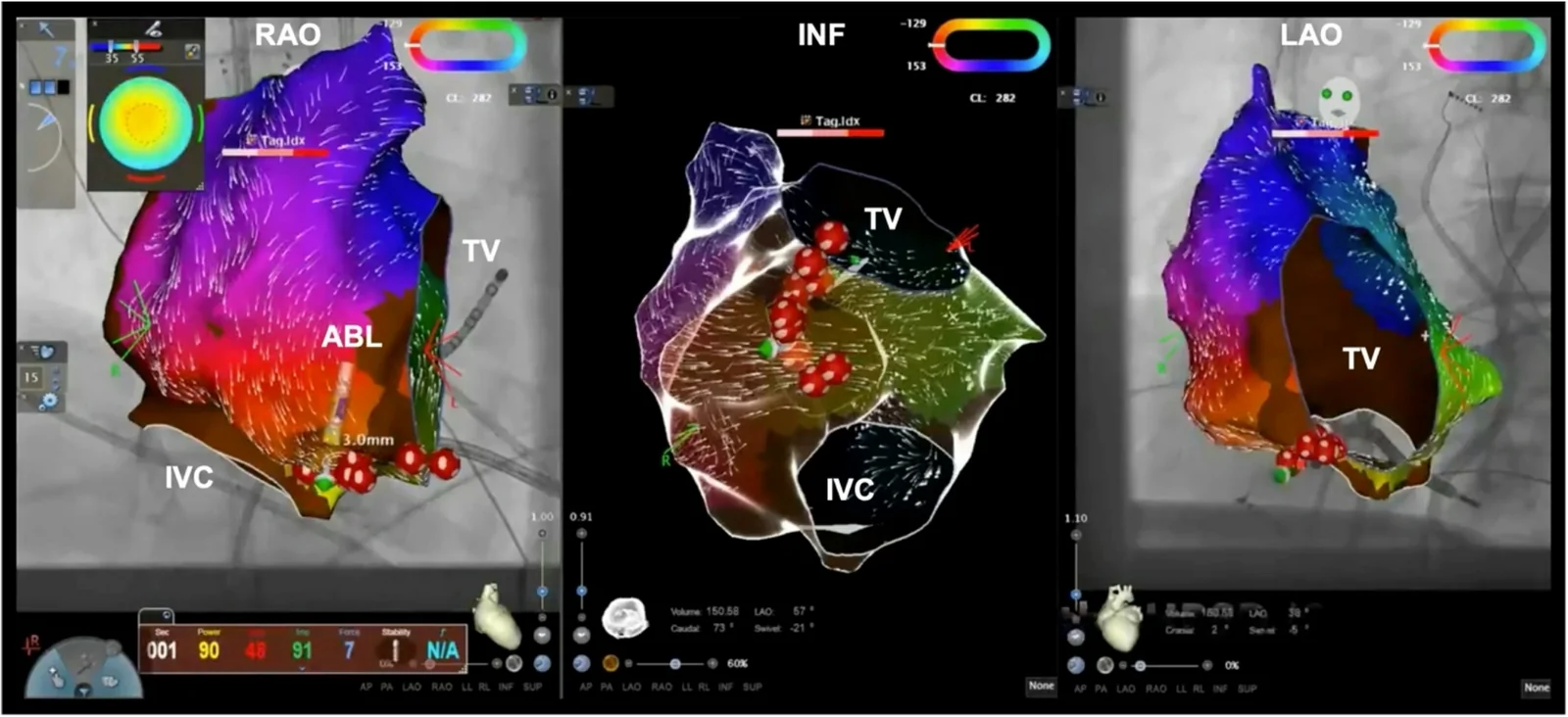
Three-dimensional electroanatomic reconstruction of the right atrium (RA) in right-anterior oblique (RAO), inferior (INF) and left-anterior oblique (LAO) views. The local activation time (LAT) map and the coherent map show typical counterclockwise atrial flutter. Red-white-dotted tags created by radiofrequency ablation catheter (ABL) with 90 watts over 4 s very high-power, short-duration ablation indicate the cavotricuspid isthmus ablation line between the tricuspid valve (TK) and inferior vena cava (IVC)

vHPSD ablation raises theoretical concerns regarding overheating and collateral injury, especially steam pops. The QDOT Micro catheter mitigates these risks through real-time temperature monitoring via six thermocouples that allow precise and dynamic power adjustment. Preclinical and early clinical data suggest vHPSD may produce less tissue edema and collateral injury compared with conventional approaches [12, 13]. In our cohort, no increase in major procedure-related complications such as tamponade, vascular injury, stroke, or steam-pop/char formation was observed. When performed carefully, vHPSD-CTI ablation appears similarly safe as AI-guided ablation protocols Fig. 1.

### Limitations

Limitations of our study include the observational, non-randomized, single-center design, modest sample size, and underrepresentation of female patients, which may introduce selection bias, as ablation strategy was determined by operator preference and catheter availability rather than random allocation. Unmeasured confounders cannot be excluded, and the study was not designed or powered to formally test non-inferiority or equivalence between ablation strategies.

A substantial proportion of patients underwent concomitant PVI for AF, which influenced overall procedure times, radiation exposure, and AT/AF/AFL recurrence. The prevalence of AF and use of AADs were higher in the vHPSD group. As antiarrhythmic therapy can occasionally organize AF into AFL, these differences may have influenced arrhythmia patterns during follow-up.

AFL recurrence rates in the vHPSD group may be underreported, as they were primarily based on Holter-ECG interpretation. Some AT recurrences in patients who did not undergo redo procedures may have represented misclassified AFL, given the absence of CTI remapping data.

Individual CTI morphology was not routinely assessed with imaging such as intracardiac echocardiography (ICE). Consequently, CTI length was not measured, which may have influenced RF delivery time and lesion number.

Formal waiting periods after achieving CTI block were generally not applied, as PVI isolation checks took precedence in combined procedures. Durability of CTI block was assessed in only a limited subset of patients undergoing redo procedures. Therefore, conclusions regarding lesion durability should be interpreted with caution and rely primarily on long-term clinical outcomes.

Despite these limitations, bidirectional CTI block was confirmed intraprocedurally, and long-term rhythm outcomes were consistently recorded. Clinical outcomes of both ablation strategies appeared comparable at 12 months, though they may further be influenced by lifestyle factors, obstructive sleep apnea, atrial size, and concomitant therapy.

Future studies, including large-scale multicenter randomized trials such as the ongoing FASD-HP trial (ClinicalTrials.gov ID: NCT05777850), dedicated CTI remapping studies, and long-term follow-up beyond 12 months, will help further assess vHPSD lesion durability and define its role in typical AFL management [23].

## Conclusions

Very-high power short-duration (vHPSD; 90 W/4 s) ablation for CTI-dependent AFL appears to be a safe and effective alternative to conventional AI/CF-guided ablation. Although vHPSD requires a higher number of RF applications, it markedly reduces CTI-RF application time while achieving consistently high rates of acute CTI block. Long-term rhythm outcomes and CTI block durability up to 12 months appear similar between vHPSD and AI-guided ablation, but durability conclusions are limited by the small number of redo procedures.

Overall, these findings complement existing evidence by extending follow-up and refining procedural characterization, while supporting the overall safety and efficiency profile of vHPSD CTI ablation in a real-world cohort, including patients undergoing concomitant AF ablation. The results should be interpreted as confirmatory and additive rather than indicative of a procedural advance.

### Clinical perspectives

1. CTI ablation with vHPSD is effective and safe, with procedural efficiency comparable to conventional protocols.
2. In compared to AI-guided CTI ablation, vHPSD requires more RF applications (inter-lesion distance 3–4 mm) but markedly reduces RF time (4x) to achieve CTI block.
3. Long-term rhythm outcomes up to 12 months and CTI block durability appear comparable to AI-guided ablation, though conclusions regarding durability are limited by the small number of patients undergoing redo procedures.

## Data Availability

Data supporting this study are curated at the Study Center of the Department of Rhythmology, University Hospital Schleswig-Holstein, Germany.

## Abbreviations

AAD: antiarrhythmic drug
AF: atrial fibrillation
AFL: atrial flutter
AI: ablation index
CA: catheter ablation
CF: contact force
CI: confidence interval
CTI: cavotricuspid isthmus
FU: follow-up
ICE: intracardiac echocardiography
ILD: inter-lesion distance
ILT: inter-lesion time
IQR: interquartile range
LA: left atrium / left atrial
PVI: pulmonary vein isolation
RA: right atrium / right atrial
RF: radiofrequency
vHPSD: very high-power short-duration

## Declarations

### Statement on usage of artifical intelligence

To ensure clear and uniform usage of proper English language ChatGPT has been used. Parts of the graphical abstract were generated using SciDraw. The output has been reviewed and edited as needed. The authors take full responsibility for the content of the publication.

### Tweet

Read & discuss about #vHPSD #90W4s for #CTI ablation.
